# Supplementary material for: A transitional species of Daspletosaurus Russell, 1970 from the Judith River Formation of eastern Montana
Source: PeerJ. 2022 Nov 25;10:e14461. doi: 10.7717/peerj.14461 (PMC9703990; doi:10.7717/peerj.14461)
Supplement: Supplemental Information 1 [file peerj-10-14461-s001.docx]

**Supplementary information for: “A new species of *Daspletosaurus* Russell, 1970 from the Judith River Formation of eastern Montana”**

Elías A. Warshaw^1, 2^ and Denver W. Fowler^2^

1. Department of Earth Sciences, Montana State University, Bozeman, Montana, USA
2. Badlands Dinosaur Museum, Dickinson, North Dakota, USA

Corresponding author:

Elías A. Warshaw^1, 2^

2137 S 11^th^ Ave 126B, Bozeman, Montana, 59715, USA

Email address: [warshawelias@gmail.com](mailto:warshawelias@gmail.com)

**Character Data**

Additions to character matrix of Carr et al. (2017):

1. **Premaxilla, orientation of alveolar margin: rostromedial-caudolateral, such that several premaxillary teeth are visible in lateral view (0); mediolateral, such that only the distalmost premaxillary tooth is visible in lateral view (1).**
2. **Maxilla, rostral interdental plates, form: first plate truncated, second plate wide (0); first plate narrow, second plate truncated (1); first two plates expanded (2) (Carr et al., 2017)*.**
3. **Lacrimal, orbital margin, form: convex (0); concave (1) (Carr et al., 2017)*.**
4. **Lacrimal, rostroventral edge of rostrodorsal ala, form: sharp, uninflated (0); forming a thick, round bar between rami that is clearly distinct from both the rostral/ventral rami and the remainder of the lacrimal antorbital fossa (1); forming a thick, round bar confluent with rostral and ventral rami but distinct from lacrimal antorbital fossa (2). ORDERED.**
5. **Quadratojugal, dorsal quadrate contact: directed medially, concealed in lateral view (0); directed caudomedially, visible in lateral view (1).**
6. **Prefrontal, orientation of long axis, dorsal view: rostrocaudal (0); rostromedial or mediolateral (1)(can be scored by the orientation of the prefrontal contact surface on the lacrimal in specimens lacking a preserved prefrontal).**
7. **Squamosal, pneumatic recess, form: not undercut around its entire margin (0); undercut around its entire margin (1) (Carr et al., 2017)*.**
8. **Parietal, joint surface for the squamosal, dorsal extent: covers the ventral half of the base of the caudolateral process (0); covers the entire caudolateral process (1) (Carr et al., 2017)*.**
9. **Prootic, tympanic ridge, presence: does not extend onto the prootic (0); extends onto the prootic (1) (Carr et al., 2017)*.**

*Proposed by Carr et al. (2017) as an autapomorphy of *D. horneri*.

Removed characters (Carr et al., 2017):

1. **Maxilla, maxillary fenestra, caudal margin, shape, lateral view: rounded (0); V-shaped (1) (Brochu, 2003; Loewen et al., 2013:34; Brusatte and Carr, 2016:327).**

The shape of the maxillary fenestra has been demonstrated to be highly variable within *T. rex*; variation in the shape of its caudal margin is taken here to be a result of individual variation (Carr, 2020; Molnar, 1990).

1. **Lacrimal, accessory caudally extending cornual process on the lateral surface between the cornual process and the supraorbital ramus, presence, lateral view: absent (0), present (1) (Carr and Williamson, 2010:70; Brusatte and Carr, 2016:360).**

We could not identify a discrete difference in the morphology of the caudal end of the rostral ramus of the lacrimal among tyrannosaurines that were scored differently for this character (i.e., *Teratophoneus* and *D. torosus, Tarbosaurus* Fig. 6 Hurum and Sabbath, 2003), and for this reason exclude this character from our analysis.

1. **Lacrimal, transition between antorbital fossa and the subcutaneous surface of the ventral ramus, form, lateral view: surfaces are continuous with each other (0); fossa is deeply inset, forming a ridge along the subcutaneous surface (1) (Carr and Williamson 2010:61; Brusatte et al., 2010:55; Brusatte and Carr, 2016:55).**

We could not identify a discrete difference in the relief between the antorbital fossa and ventral ramus of the lacrimal in taxa scored for (0) and (1) (i.e., *Bistahieversor* and *D. torosus*) and for this reason exclude this character from our analysis.

1. **Quadratojugal, jugal articulation, form, lateral and dorsal views: dorsal quadratojugal process of jugal does not (0) or does (1) approach the base of the quadratojugal (caudoventral corner of the laterotemporal fenestra) (Carr and Williamson, 2010:118; Brusatte et al., 2010:100; Loewen et al., 2013:146; Brusatte and Carr, 2016:100).**

We could not identify a discrete difference between the morphology of the dorsal jugal articulation in taxa scored for (1) or (0), and for this reason exclude this character from our analysis.

1. **Pes, metatarsal II, lateral surface, shape, proximal view: flat or weakly concave (0); strongly concave (1) (Brusatte et al., 2010:304; Loewen et al., 2013:486; Brusatte and Carr, 2016:304).**

This character is redundant with the presence of an arctometatarsalian metatarsus (character 377) and is for this reason excluded from our analysis.

Modified characters (Carr et al., 2017):

1. **Lacrimal, cornual process, height, lateral view: tall, greater than 60% height of antorbital fossa below it (0); short, less than 60% of the height of the fossa below it (1) indistinguishable in relief from the rest of the dorsal surface of the lacrimal (2)(var. Loewen et al., 2013:64; Brusatte and Carr, 2016:359). ORDERED.**

An additional character state has been added here to distinguish the morphology of the lacrimal’s cornual process in *Tyrannosaurus* and *Tarbosaurus* as opposed to more basal tyrannosaurines, including *D. horneri*. Otherwise, we agree with the scorings of Carr et al. (2017), but we note here that the usage of the antorbital fossa as a reference against which to score the height of the cornual process is potentially misleading since the height of the antorbital fossa is itself quite variable between taxa (i.e., CMN 8506, *D. torosus*, vs. MOR 590, *D. horneri*). *D. wilsoni* is scored as having a short cornual process of the lacrimal based on comparisons of the absolute height of the process with *Daspletosaurus* specimens of other species (see main article text).

1. **Postorbital, cornual process, presence and form, lateral view: absent (0); limited to rugose rim or small rugose convexity at caudodorsal corner of orbit (1); swollen such that the process overhangs its caudoventral margin, forming a crease between the process and the main body of the postorbital (2); entirety of the caudodorsal region of the postorbital inflated, with cornual process expressed as a large, convex boss that grades into the body of the postorbital caudoventrally (3) (Holtz, 2001:56; Sereno et al., 2009:29; Brusatte et al., 2010:81; Loewen et al., 2013:110). ORDERED.**

This character is modified to distinguish between the cornual process morphologies of *D. torosus* and *D. horneri* + *Tyrannosaurus*.

1. **Frontal, contribution to orbital rim, dorsal and lateral views: extensive (0), present but limited to a small notch (1), excluded by postorbital-lacrimal contact in large specimens (2), ~~excluded by postorbital-lacrimal articulation and novel palpebral ossification (3)~~ (Sereno et al., 2009:21; Carr and Williamson, 2010:104; Brusatte et al., 2010:120; Loewen et al., 2013:90; Brusatte and Carr, 2016:120). ORDERED.**

State 3 is redundant with character #148 of Carr et al., (2017) (presence of a palpebral ossification), and is for this reason collapsed into state 2.

Modified character states (Carr et al., 2017):

1. ***D. horneri* (? → 2)**

Given the relatively complete orbitotemporal region of the holotype of *D. horneri*, we feel comfortable assigning the shape of its orbit to character state 2 (dorsoventrally tall, more than twice as tall as long).

1. ***D. horneri* (0 → 1)**

The jugal of *D. horneri* flexes rostromedially at the level of the orbit, directing the orbits forwards as in *T. rex*, such that their orientation is best described by character state 1 (directed rostrally at greater than 20 degrees from the sagittal plane).

1. ***D. horneri* (0 → 1)**

Personal observation of the holotype specimen of *D. horneri* (MOR 590) by EW revealed that the maxillary fenestra abuts the ventral margin of the antorbital fossa in this specimen.

1. ***D. horneri* (0 → 1)**

Personal observation of the holotype specimen of *D. horneri* (MOR 590) by EW revealed that the antorbital fossa does not extend ahead of the rostral boundary of the external antorbital fenestra in this specimen.

1. ***A. libratus*, *A. sarcophagus*, *Teratophoneus*, and *Qianzhousaurus* (2 → 1)**

We regard the morphology of the postorbital’s cornual process in the taxa listed above as best represented by character state 1 (i.e., a small rugose convexity at the orbit’s caudodorsal margin) as opposed to 2 (a massively swollen tuberosity), and sought to distinguish the postorbital morphology of these taxa from the much more prominent cornual processes of derived tyrannosaurines (character states 2 and 3).

1. ***D. horneri* (1 → 0)**

We disagree with Carr et al.’s (2017) characterization of the postorbital cornual process of *D. horneri* as nearing the laterotemporal fenestra (as is the case in *D. torosus*); we regard the position of this process as more similar to the condition in *T. rex*: widely separated from the laterotemporal fenestra and terminating more closely to the orbit.

1. ***D. horneri* (1 → 0)**

We disagree with Carr et al.’s (2017) characterization of the suborbital fenestra of *D. horneri* as rostrocaudally elongate; personal observation of the articulated skull of the holotype specimen of this species (MOR 590) by EW revealed a subcircular fenestra similar to most specimens of *T. rex*.

***Daspletosaurus* autapomorphies recovered by the cladistic analysis:**

*D. horneri*: 89 (0 → 1), 96 (0 → 1), 158 (1 → 0), 206 ( 1 → 0), 275 (1 → 0), 305 (2 → 1)

*D. torosus*: 89 (0 → 1), 272 (0 → 1), 364 (1 → 0)

*D. wilsoni*: 251 (2 → 1)

**Synapomorphies recovered by the cladistic analysis:**

*Daspletosaurus* + more derived tyrannosaurines: 20 (0 → 1), 24 (0 → 2), 42 (1 → 2), 43 (0 → 2), 59 (0 → 1), 75 (0 → 1), 76 (1 → 0), 78 ( 1 → 2), 85 (0 → 1), 87 (0 → 1), 91 (0 → 1), 93 (0 → 1), 97 (0 → 1), 110 (1 → 2), 111 (1 → 2), 117 (0 → 1), 127 (0 → 1), 160 (1 → 2), 220 (0 →1), 231 (0 → 2), 232 (0 → 1), 238 (0 → 1), 361 (1 → 2), 384 (0 → 1)

*D. wilsoni* + more derived tyrannosaurines: 7 (1 → 2), 69 (0 → 1), 395 (0 → 1)

*D. horneri* + more derived tyrannosaurines: 9 (0 → 1), 110 (2 → 3), 396 (0 → 1), 400 (0 → 1)

**Supplemental Figures**

**
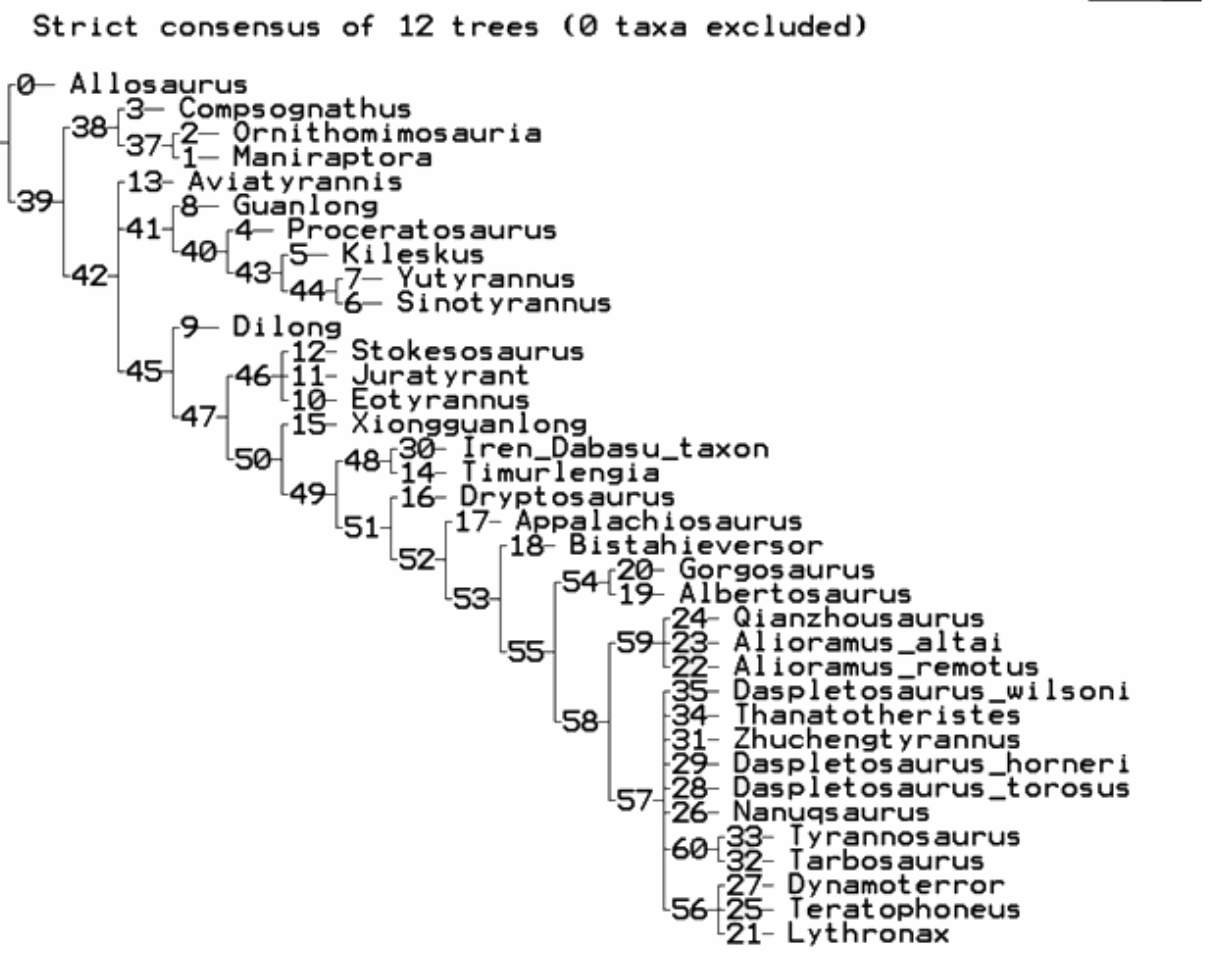
**

**Figure S1: Strict consensus of cladistic analysis with all taxa included.** Note the large polytomy formed by tyrannosaurines more derived than alioramini (node 57). See main text for source of matrix and settings used for analysis.
